# Potential risk of proton pump inhibitors for Parkinson’s disease: A nationwide nested case-control study

**DOI:** 10.1371/journal.pone.0295981

**Published:** 2023-12-14

**Authors:** Ji Taek Hong, Hye-Kyung Jung, Kwang Jae Lee, Eun Jeong Gong, Cheol Min Shin, Jong Wook Kim, Young Hoon Youn, Bora Lee

**Affiliations:** 1 Department of Internal Medicine, College of Medicine, Ewha Womans University, Seoul, Republic of Korea; 2 Department of Gastroenterology, Ajou University School of Medicine, Suwon, Gyeonggi-do, Republic of Korea; 3 Department of Internal Medicine, Gangneung Asan Hospital, University of Ulsan College of Medicine, Gangneung, Gangwon-do, Republic of Korea; 4 Department of Internal Medicine, Seoul National University Bundang Hospital, Seongnam, Gyeonggi-do, Republic of Korea; 5 Department of Internal Medicine, Inje University Ilsan Paik Hospital, Goyang, Republic of Korea; 6 Department of Internal Medicine, Gangnam Severance Hospital, Yonsei University College of Medicine, Seoul, Republic of Korea; 7 Institute of Health and Environment, Seoul National University, Seoul, Republic of Korea; 8 RexSoft Corp., Seoul, Republic of Korea; Chung Shan Medical University, TAIWAN

## Abstract

Proton pump inhibitor (PPI) use is a potential risk factor for neurodegenerative disease development; however, its role in Parkinson’s disease (PD) remains unclear. This study aimed to investigate the association between PPI use and PD risk. A total of 31,326 patients with newly diagnosed PD were matches by age, sex, body mass index, diabetes, and hypertension with 125,304 controls at a ratio of 1:4. The data were collected from the Korean National Health Insurance Services Database from January 2010 to December 2019. Cumulative defined daily doses of PPIs were extracted from treatment claims. We examined the association between PPI use and PD risk using conditional logistic regression. To prevent protopathic bias, we excluded patients diagnosed with PD within a 1-year lag period after PPI exposure. We applied 2- and 3-year lag periods for sensitivity analysis. PPI use was associated with an increased risk of PD when a 1-year lag period was applied between PPI exposure and PD development (adjusted odds ratio, 1.10; 95% confidence interval, 1.07–1.13). A significant positive dose-response relationship existed between the cumulative defined daily doses of PPIs and PD development (*P*<0.001). Similar results were obtained for the 2- or 3-year lag periods. The association did not vary based on gender. Older age, a higher Charlson Comorbidity Index score, no alcohol consumption, and a non-smoking status were associated with a significantly increased PD risk with PPI use. We observed an association between PPI use and PD risk, although long-term follow-up studies are necessary to verify this association.

## Introduction

Parkinson’s disease (PD), a chronic neurodegenerative disorder, is characterized by motor symptoms, such as tremors, bradykinesia, and rigidity, and a wide range of non-motor symptoms that affect many domains [1]. Similar to other progressive neurodegenerative disorders, intracellular protein deposition in patients with PD is a characteristic finding that is crucially attributed to a protein removal defect resulting from lysosomal dysfunction [2]. However, the underlying mechanism of PD remains poorly understood. PD is associated with various causes, including complex interactions between genetic and environmental factors. In addition, PD has an increasing global burden owing to the increasing older population and potential impact of environmental factors, which are expected to further increase in the future [3]. Therefore, identifying potentially modifiable risk factors for PD is indispensable.

Proton pump inhibitors (PPIs) are most commonly used to treat gastroesophageal reflux disease and ulcers and can pass through the blood-brain barrier (BBB) [4]. Their use contributes to the pathogenesis of neurodegenerative diseases by inhibiting lysosomal acidification through the inhibition of vacuolar proton pumps and by preventing the degradation ability of fibrillar amyloid-β (Aβ), an Aβ degradation product [5]. Therefore, PPI exposure may be a risk factor for various neurodegenerative diseases. Previous studies [6–8] have not demonstrated a relationship between PPI use and the risk of dementia, Alzheimer’s disease, and amyotrophic lateral sclerosis. Few epidemiological studies [9–11] have reported an association between PD and PPI use. However, these studies have some limitations. The dose-response relationship is unclear, and more prolonged effects are also not known. In practice, there are growing concerns reported regarding the potential abuse and use of PPIs for longer periods than recommended in clinical guidelines due to inappropriate indications. Additionally, identifying high-risk groups for PD among PPI users is important for primary prevention. Therefore, we aimed to examine the relevance of PPI use as a risk factor for PD and the dose–response relationship. Given the wide use of PPIs and that PD is one of the most serious diseases to date, the confirmation of these results is likely to be significant.

## Materials and methods

### Data source

Data were extracted from the population-based cohort of the Korean National Health Insurance Service (NHIS) database, which covers approximately 97% of the Korean population, is managed by the Korean government, and comprises > 95% of all national healthcare facilities. We used the 2002–2019 NHIS data in this study. Data were accessed on 4th December, 2020, for research purposes. The National Health Insurance recommends members to undergo a comprehensive health checkup at least once every 2 years, with changing recommendations based on each individual’s age. The Institutional Review Board of the author institute approved this study (approval number: EUMC 2020-07-028) and waived the requirement for obtaining informed consent because the database maintained de-identified and anonymous data of sampled individuals. This study also complied with the 1964 Declaration of Helsinki and its later amendments or comparable ethical standards.

### Study design

**Fig 1:**
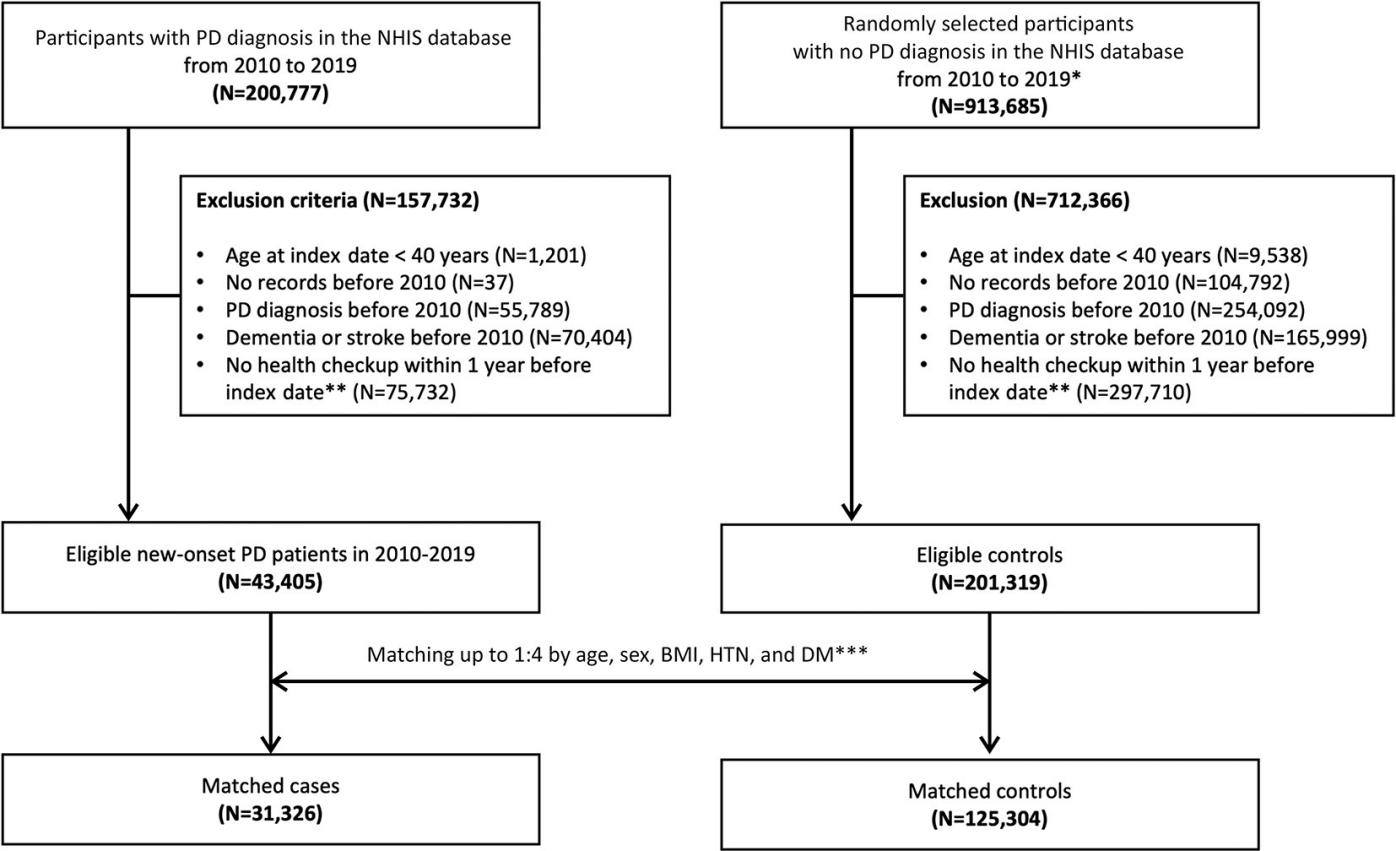
Flowchart of the study population. *Among those without a PD diagnosis from 2010 to 2019, participants with the same age and sex at the index date as those with a PD diagnosis were randomly selected in quadruplicates. **The index date was defined as the first date of a PD diagnosis as the disease code recorded in the NHIS database. ***Variables were measured at 5 years before the index date. Abbreviations: BMI, body mass index; DM, diabetes mellitus; HTN, hypertension; NHIS, the Korean National Health Insurance Service; PD, Parkinson’s disease.

A nested case-control study was designed with cases that had been diagnosed as PD between 2010 and 2019 (N = 200,777). Controls comprised participants with no PD diagnosis between 2010 and 2019. Under the regulation of the NHIS institute, the total control population should be reduced to four times that of the cases. Thus, the initial population of controls was randomly selected from the whole control cohort between the aforementioned period, as stratified by age and sex on the index date (N = 913,685). The index date was defined as the first date of PD diagnosis. After applying the exclusion criteria, cases (N = 43,405) and controls (N = 201,319) were matched in a ratio of 1:4 based on age, sex, body mass index (BMI), hypertension, and diabetes mellitus at 5 years before the index date. Finally, 31,326 matched cases and 125,304 matched controls were obtained (Fig 1).

### Identification of PD

Between 2010 and 2019, we defined patients with newly diagnosed PD as those meeting at least one of the following criteria:

1. Based on the *International Classification of Diseases*, *10*^*th*^ *Revision*, *Clinical Modification* (ICD-10-CM) criteria, the G20 code was utilized at least once among the available diagnostic codes, including the primary and sub-diagnosis codes, and PD drugs were prescribed for ≥60 days (S1 Table) [12].
2. More than one diagnosis on the PD registration code (V124) in the Intractable Diseases Registry.

The first definition was formulated according to that in previous studies [13–15] and the expert opinion of a neurologist. PD drugs were also determined based on the expert opinion of a neurologist [16]. Among all drugs, we excluded those that can be used for indications other than PD, including amantadine, apomorphine, benzatropine, biperiden, bromocriptine, cabergoline, dihydroergocryptine, orphenadrine, piribedil, procyclidine, rotigotine, and trihexyphenidyl. Finally, we included the following PD drugs (in the format of Anatomical Therapeutic Chemical (ATC) classification system code [drug name]): N04BX02 (entacapone); N04BA02 and N04BA03 (levodopa combinations); N04BC02 (pergolide); N04BC05 (pramipexole); N04BD02 (rasagiline); N04BC04 (ropinirole); and N04BD01 (selegiline), which are specifically used for PD treatment (S1 Table) [17].

The second definition is very similar to the United Kingdom Parkinson’s Disease Society Brain Bank criteria for the V124 code used for clinical and research purposes [18]. We excluded patients diagnosed with PD before 2010 and those diagnosed with dementia (ICD-10 codes: F00, F01, F02, F03, G30, G31, and G32) or cerebrovascular disease (ICD-10 codes: I60-I69, G45-G46), which could lead to misclassification. Based on the definition of PD, we defined the index date of PD as the date of the first G20 diagnosis, the date of the first PD drug prescription (whichever came first), or the date of the first diagnosis of the V124 code. Patients with PD and controls (in a 1:4 ratio) were matched on the index date for age, sex, BMI, hypertension, and diabetes mellitus. Moreover, we calculated the Charlson Comorbidity Index (CCI) score based on the 5 years before the index date.

According to a study in Korea that investigated prevalence using the same definition as the one used in this study, the average prevalence of PD between 2012 and 2015 was 171.01 per 100,000 persons. The average age-standardized prevalence of PD according to the WHO 2000–2025 and US 2000 standard population between 2012 and 2015 was 114.13 and 176.21 per 100,000 persons, respectively [16]. Thus, the study showed a similar prevalence rate to existing worldwide data.

### PPI exposure

PPI use was based on A02BC ATC codes (drug name) as follows: A02BC01 (omeprazole), A02BC02 (pantoprazole), A02BC03 (lansoprazole), A02BC04 (rabeprazole), A02BC05 (esomeprazole), A02BC06 (dexlansoprazole), and A02BX (ilaprazole) [19]. Information regarding all PPIs dispensed between January 1, 2010 and the index date was extracted from the Prescribed Drug Register. PPI use was defined as at least one PPI prescription during the study period. PPIs are not over-the-counter drugs in Korea; therefore, all drugs categorized as PPIs are registered in this database. The cumulative defined daily dose (cDDD) for PPIs was calculated as the total dispensed number of defined daily doses between January 1, 2010 and the index date. Therefore, we initially defined PPI use as a categorical variable with two groups (i.e., PPI users and non-users). PPI users were subsequently further subdivided into three groups based on the exposure amount and tertile distribution of cDDD as follows: (1) very low exposure (<180 DDDs); (2) low exposure (180–359 DDDs); and (3) high exposure (≥360 DDDs). We employed 1- to 3-year lag windows before the index date for sensitivity analyses.

### Covariates for risk adjustment

The PD-related variables included diabetes mellitus [20], hypertension [21], dementia [22], stroke [23], smoking [24], alcohol use [25], and BMI [26]. Excluding the variable used for matching, the following four variables were included as potential confounders: calendar year of the index date, CCI, smoking status, and alcohol consumption. Smoking status and alcohol consumption were extracted from the records of health examinations and categorized as follows: smoking status, never smoker, ex-smoker, or current smoker; and alcohol consumption, none, rarely (1–2 times/week), or frequently (≥3 times/week). Additionally, disease status was assessed through the modified CCI proposed by Sundararajan et al., [27] calculated as presented in S2 Table.

### Statistical analysis

Descriptive statistics are presented as means (standard deviations [SDs]) or medians (interquartile ranges) for continuous variables and frequency (percentage) for categorical variables. Group comparison was conducted using the two-sample *t*-test or Mann–Whitney *U* test for continuous variables based on the normality test results or chi-square or Fisher’s exact test for categorical variables, as appropriate. We analyzed the data under a case-control design to compare the use and dose of PPIs between patients with PD and healthy controls. Conditional logistic regression was conducted to determine the association between PPI exposure and PD with adjustment for covariates excluding the matching variables (i.e., calendar year of the index date, CCI, smoking status, and alcohol consumption). The odds ratios (ORs) and their 95% confidence intervals (CIs) represented the degree of association. Only PPI use that occurred within the window of 5 years before PD diagnosis was considered in the analysis, and the lag time was set at 1, 2, and 3 years before PD diagnosis for sensitivity analyses. A two-tailed p-value of <0.05 was considered statistically significant. All analyses were conducted using SAS version 9.4 (SAS Institute, Cary, NC, USA) and R version 4.1.2 (R Foundation for Statistical Computing, Vienna, Austria).

## Results

### Baseline characteristics

Table 1 summarizes the baseline characteristics of the included participants, who were well-matched between the groups. The cohort’s mean age (SD) was 67.65 (8.85) years, and 44.56% of patients were male. Regarding diseases considered as risk factors, a higher proportion of individuals with a CCI score of ≥3 points was observed in the PD group.

**Table 1:** Characteristics of the study population.

Variable	Matched cases	Matched controls	p-value
	(N = 31,326)	(N = 125,304)	
**Age (years) at index date, mean±SD**	67.72±8.92	67.63±8.83	0.134
**Female, n (%)**	17,366 (55.44%)	69,464 (55.44%)	1
**BMI category (kg/m** ^**2**^ **), n (%)**			1
Underweight (<18.5)	830 (2.65%)	3,320 (2.65%)	
Normal (18.5–24.9)	19,133 (61.08%)	76,536 (61.08%)	
Overweight (25.0–29.9)	10,018 (31.98%)	40,072 (31.98%)	
Obesity (≥30.0)	1,344 (4.29%)	5,376 (4.29%)	
2018–2019	1,543 (4.93%)	5,465 (4.36%)	
**Comorbidities, n (%)**			
HTN	200 (0.64%)	800 (0.64%)	1
DM	68 (0.22%)	272 (0.22%)	1
**Calendar year of the index date, n (%)**			<0.001
2010–2011	5,948 (18.99%)	26,649 (21.27%)	
2012–2013	7,063 (22.55%)	28,796 (22.98%)	
2014–2015	8,401 (26.82%)	32,303 (25.78%)	
2016–2017	8,370 (26.72%)	32,091 (25.61%)	
2018–2019	1,543 (4.93%)	5,465 (4.36%)	
**CCI score group, n (%)**			<0.001
0	9,318 (29.75%)	56,032 (44.72%)	
1	9,393 (29.99%)	36,993 (29.52%)	
2	6,274 (20.03%)	18,090 (14.44%)	
≥3	6,340 (20.24%)	14,189 (11.32%)	
**Smoking status, n (%)**			<0.001
Never	23,650 (75.50%)	87,961 (70.20%)	
Ex-/current smoker	7,675 (24.50%)	37,343 (28.98%)	
**Alcohol consumption (times/week), n (%)**			<0.001
None	25,280 (80.70%)	88,994 (71.02%)	
1–2	4,058 (12.95%)	21,496 (17.16%)	
≥3	1,987 (6.34%)	14,814 (11.82%)	

Abbreviations: BMI, body mass index; CCI, Charlson Comorbidity Index; DM, diabetes mellitus; HTN, hypertension

### PPI exposure and PD risk

Table 2 illustrates the odds of PPI exposure in PD. PPI users comprised 15,467 (49.4%) patients with PD and 55,407 (44.2%) healthy controls. PPI use was associated with an increased risk of PD (adjusted OR, 1.10; 95% CI, 1.07–1.13) after applying a 1-year lag period. We observed a significant positive dose-response relationship between the cDDDs of PPIs and PD development (p for trend <0.001), in which the highest dose-response effect was demonstrated among patients with a PPI exposure of ≥360 cDDD (adjusted OR, 1.36; 95% CI, 1.26–1.47). The results were similar after employing 2-year and 3-year lag windows.

**Table 2:** Association between PPI use and PD risk.

Category	Including a lag window of 1 year		Including a lag window of 2 years		Including a lag window of 3 years	
	Cases/controls	OR† (95% CI)*	Cases/controls	OR† (95% CI)	Cases/controls	OR† (95% CI)
**Whole population (N = 156,629)**						
PPI non-user	15,858/69,897	1 (reference)	18,517/80,054	1.00 (reference)	21,817/91,973	1 (reference)
PPI user	15,467/55,407	1.10 (1.07–1.13)***	12,808/45,250	1.11 (1.07–1.14)***	9,508/33,331	1.09 (1.06–1.13)***
By cDDD						
0	15,858/69,897	1 (reference)	18,517/80,054	1.00 (reference)	21,817/91,973	1 (reference)
1–179	12,529/47,518	1.06 (1.03–1.10)***	10,793/39,875	1.08 (1.05–1.11)***	8,323/30,103	1.08 (1.04–1.11)***
180–359	1,448/4,180	1.29 (1.20–1.39)***	1,080/3,024	1.27 (1.16–1.38)***	680/1,934	1.23 (1.10–1.37)***
≥360	1,490/3,709	1.36 (1.26–1.47)***	935/2,351	1.33 (1.21–1.47)***	505/1,294	1.26 (1.11–1.43)***
p for trend		<0.001		<0.001		<0.001
**Male (n = 69,799)**						
PPI non-user	7,368/31,897	1.00 (reference)	8,523/36,254	1.00 (reference)	9,979/41,374	1 (reference)
PPI user	6,591/23,943	1.06 (1.01–1.11)*	5,436/19,586	1.07 (1.02–1.12)**	3,980/14,466	1.04 (0.99–1.10)
By cDDD						
0	7,368/31,897	1.00 (reference)	8,523/36,254	1.00 (reference)	9,979/41,374	1 (reference)
1–179	2,513/10,163	1.03 (0.98–1.08)	2,243/8,781	1.05 (1.00–1.10)	1,758/6,938	1.03 (0.98–1.09)
180–359	1,972/7,491	1.24 (1.10–1.40)***	1,678/6,231	1.19 (1.04–1.37)*	1,275/4,643	1.04 (0.88–1.24)
≥360	920/3,037	1.23 (1.08–1.39)*	701/2,349	1.23 (1.06–1.43)**	477/1,543	1.30 (1.06–1.59)*
p for trend		<0.001		<0.001		<0.001
**Female (n = 86,830)**						
PPI non-user	8,490/38,000	1.00 (reference)	9,994/43,800	1.00 (reference)	11,838/50,599	1 (reference)
PPI user	8,876/31,464	1.13 (1.09–1.18)***	7,372/25,664	1.14 (1.09–1.18)***	5,528/18,865	1.13 (1.09–1.18)***
By cDDD						
0	8,490/38,000	1.00 (reference)	9,994/43,800	1.00 (reference)	11,838/50,599	1 (reference)
1–179	3,408/13,634	1.09 (1.05–1.14)***	3,109/11,788	1.10 (1.06–1.15)***	2,513/9,340	1.11 (1.06–1.16)***
180–359	2,510/9,130	1.33 (1.20–1.46)***	2,126/7,660	1.33 (1.19–1.49)***	1,644/5,616	1.37 (1.19–1.57)***
≥360	1,206/4,063	1.45 (1.32–1.61)***	936/3,066	1.40 (1.24–1.59)***	656/2,023	1.24 (1.06–1.46)**
p for trend		<0.001		<0.001		0.273
**Age ≥50 years (n = 105,194)**						
PPI non-user	15,159/66,855	1.00 (reference)	17,722/76,713	1 (reference)	20,913/88,317	1 (reference)
PPI user	15,056/54,009	1.10 (1.07–1.13)***	12,493/44,151	1.11 (1.07–1.14)***	9,302/32,547	1.10 (1.06–1.13)***
By cDDD						
0	15,159/66,855	1.00 (reference)	17,722/76,713	1 (reference)	20,913/88,317	1 (reference)
1–179	5,675/22,965	1.06 (1.03–1.10)***	5,159/19,882	1.08 (1.05–1.12)***	4,138/15,764	1.08 (1.04–1.12)***
180–359	4,377/16,210	1.29 (1.20–1.39)***	3,724/13,588	1.27 (1.17–1.39)***	2,872/10,045	1.23 (1.10–1.37)***
≥360	2,095/7,006	1.36 (1.26–1.47)***	1,614/5,352	1.34 (1.21–1.47)***	1,119/3,529	1.26 (1.11–1.43)***
p for trend		<0.001		<0.001		<0.001
**Age <50 years (n = 151,079)**						
PPI non-user	699/3,042	1.00 (reference)	795/3,341	1 (reference)	904/3,656	1 (reference)
PPI user	411/1,398	1.09 (0.92–1.28)	315/1,099	1.00 (0.84–1.19)	206/784	0.94 (0.77–1.16)
By cDDD						
0	699/3,042	1.00 (reference)	795/3,341	1 (reference)	904/3,656	1 (reference)
1–179	246/832	1.08 (0.92–1.28)	193/687	1.00 (0.84–1.19)	133/514	0.93 (0.75–1.14)
180–359	105/411	1.15 (0.60–2.21)	80/303	1.03 (0.50–2.09)	47/214	1.19 (0.49–2.85)
≥360	31/94	1.34 (0.49–3.62)	23/63	1.08 (0.27–4.32)	14/37	3.13 (0.26–38.13)
p for trend		0.005		0.137		0.418

Abbreviations: cDDD, cumulative defined daily dose CI, confidence interval; OR, odds ratio; PPI, proton pump inhibitor

*, p<0.05

**, p<0.01

***, p<0.001

**†** adjusted for the calendar year of the index date, Charlson Comorbidity Index score, smoking status, and alcohol consumption.

### Subgroup analyses of the association between PPI use and PD risk

**Fig 2:**
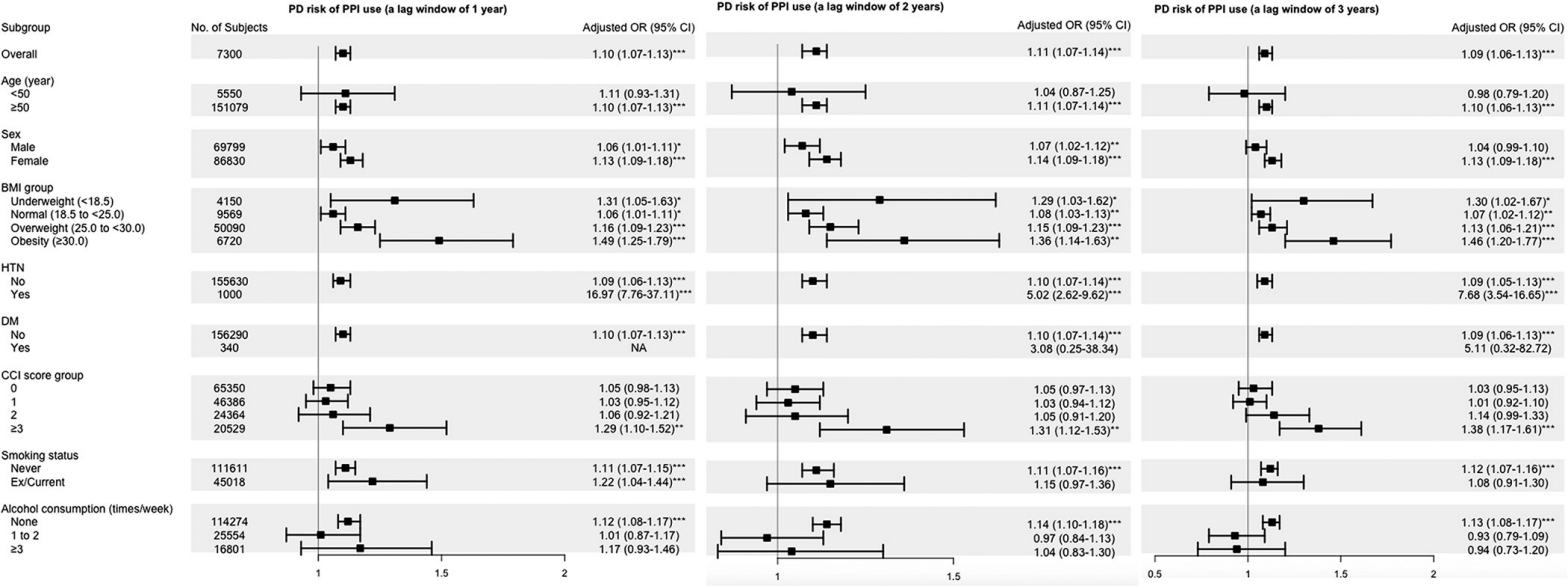
Adjusted odds ratios with 95% confidence intervals for Parkinson’s disease risk stratified by various clinical variables. The OR and 95% CI were adjusted for the calendar year of the index date, CCI score, smoking status, and alcohol consumption. Abbreviations: BMI, body mass index; CCI, Charlson Comorbidity Index; CI, confidence interval; OR, odds ratio.

We also conducted subgroup analyses for the association between PPI use and PD risk. Subgroup analysis revealed that the PD risk with PPI use was higher in female than in male participants. PPI use increased the risk of PD in patients aged ≥50 years (OR, 1.10; 95% CI, 1.07–1.13); however, there was no significant risk in individuals aged <50 years (OR, 1.11; 95% CI, 0.93–1.31). PPI use was also associated with a significantly increased risk of PD in patients who did not consume alcohol rather than in those who consumed alcohol (1–2 times/week or ≥3 times/week). Further, PPI use was associated with a significantly increased risk of PD in individuals with a CCI score of ≥ 3 points compared with that in individuals with a CCI score of 0, 1, or 2 points as well as in non-smokers compared with that in ex- or current smokers (Fig 2 and S3 Table).

## Discussion

In this nested case-control study, we evaluated the relationship between PPI use and PD risk using a Korean nationwide population-based dataset. Our results have demonstrated an association between PPI use and PD risk after applying a 2-year or 3-year lag window before diagnosis, with evidence of a dose-response relationship. Furthermore, older individuals (age ≥50 years) were more susceptible to the PPI-related risk of PD. Several hypotheses could explain our findings.

First, protopathic bias may exist because the agent of interest is used to treat the first symptom of an event. Patients with PD present with increased gastrointestinal dysfunction, which may have begun years before their PD diagnosis [24]. They may exhibit various symptoms, including nausea, abdominal bloating, constipation, early dyspepsia, upper abdominal pain, and weight loss, due to dysfunction of the PD-related autonomic and enteric nervous systems. Furthermore, abdominal symptoms of preclinical PD may increase hospitalization and clinic visits, leading to increased PPI use. Considering the characteristics of our patient group, a higher proportion of individuals with a CCI score of ≥1 points was observed in the PD group, and that group may have had increased PPI use. To reduce this bias, we applied various lag periods for the sensitivity analysis with adjustments for the CCI score. All analyses revealed a significant relationship between PPI use and PD risk.

The second hypothesis is related to the lipophilic properties of PPIs and their ability to cross the BBB. PPIs may affect Aβ metabolism, one of the pathological markers of Alzheimer’s disease. As PPIs can cross the BBB, Aβ degradation and deposition decrease and increase in the brain, respectively. Recent evidence has increasingly demonstrated the relevance of Aβ in PD and its possible role in related cognitive deficits [28]. PPI may also induce changes in the cholinergic system. A recent study [29] reported that it might limit the action of choline-acetyltransferase, which has an important role in the biosynthesis of cholinergic signaling substances, and damage to cholinergic interneurons caused by this action can lead to movement disorders, including PD.

Third, other possible mechanisms in the relationship between PPI use and PD risk could involve magnesium, vitamin B12, and iron deficiencies. Long-term PPI use may decrease the secretion of gastric acid, resulting in magnesium deficiency and decreased vitamin B12 binding protein levels, which may impair vitamin B12 and iron absorption [30]. A clinical study [31] reported that magnesium, via its neuroprotective action, reduces lipid peroxidation and regulates oxidative stress by inhibiting the generation of free radicals. Therefore, magnesium deficiency may increase calcium influx and cause neurotoxicity, thus leading to impaired neuronal function [31]. Decreased gastric acid secretion decreases acidity in the small intestine, leading to bacterial overgrowth and malabsorption [32]. Recent studies [33, 34] have reported a relationship between vitamin B12 deficiency and PD risk. Moreover, patients with PD have lower vitamin B12 levels than healthy individuals; low vitamin B12 levels are associated with peripheral neuritis, cognitive impairment, and accelerated disease progression [34]. A study [35] that used iron-deficient anemic mouse models showed that iron deficiency can impair dopamine reuptake because tyrosine hydroxylase, which is iron-dependent, is important for dopamine synthesis.

Another possible mechanism is gut microbiota imbalance. PPIs can induce changes in gut microbiota composition; these changes have been confirmed in the stool samples of patients with PD compared with those of healthy controls [36]. Moreover, another study [37] demonstrated that the production of short-chain fatty acids, major metabolites of specific intestinal bacteria, decreased in patients with PD compared with that in controls, suggesting gut microbiota dysbiosis [37]. Consequently, this gut microbiota dysbiosis may be associated with pathophysiological changes in the gastrointestinal, enteric nervous, and central nervous systems.

Recent studies have not reported a clear relationship between PPI use and dementia, Alzheimer’s disease, and amyotrophic lateral sclerosis. However, we observed a significant relationship between PPI use and PD risk. Few studies have investigated the effect of PPI therapy on PD risk [9–11]. A study compared 3,026 patients with PD and 12,104 controls; comparison of current users (PPI usage time was 1 month before the index date) and past users (PPI usage time was 31–365 days before the index date), with ORs of 1.63 (95% CI, 1.44–1.84) and 1.12 (95% CI, 1.01–1.25), respectively, indicated a significant association between PPI use and PD risk. However, the aforementioned study only covered exposure to PPIs over a 1-year period; therefore, the long-term effects of PPIs on PD were not determined. In our study, we investigated the long-term effect by analyzing PPI use for 5 years and employing different lag periods. A case-control study [9] that compared patients with PD and controls (each group, N = 4,280) aged ≥65 years reported a significant relationship between PD and PPI use (OR, 1.15; 95% CI, 1.04–1.27). The results of the aforementioned study were also significantly associated with older age, which is consistent with our results. Similarly, a Danish study [10] that examined the relationship between PD and prior treatment for *Helicobacter pylori* infection showed an increased risk of PD among patients who were treated with PPIs alone based on a 0-year lag (OR, 1.26; 95% CI, 1.16–1.36) and 5-year lag (OR, 1. 23; 95% CI, 1.11–1.37) before PD diagnosis; the risk estimates were not modified by age stratification. However, in our study, subgroup analysis revealed age differences in the relationship between PPI use and PD risk. Furthermore, owing to differences in the clinical picture, progression, and response to drugs and based on the symptom onset period, PD can be classified as juvenile, young-onset (YOPD), and late-onset PD [38]. The onset age of YOPD is between 21 and 40–50 years [38]. We conducted a subgroup analysis with a 50-year-old cut-off value to investigate the effect of PPI use on YOPD. Our results show a significant relationship between PPI use and PD risk in patients aged ≥50 years but not in patients aged <50 years. Theoretically, genetic and environmental factors may be significantly involved in YOPD and sporadic PD, respectively. The onset age in PD is negatively associated with the risk of a genetic predisposition [39]; thus, PPI use is not associated with YOPD risk, which strongly involves genetic factors.

Furthermore, subgroup analyses were conducted to determine the relationship between PPI use and PD risk. Identifying high-risk groups for PD among PPI users is important for primary prevention (Fig 2 and S3 Table). As previously mentioned, PPI use significantly increased PD risk in patients aged ≥50 years; however, there was no increased risk in patients aged <50 years. In contrast, sex and BMI did not modify PD risk in PPI users. These results are similar to those of previous studies regarding the risk factors for PD. Many longitudinal studies identified no association between BMI and PD risk [40, 41]; however, the lack of an association is inconclusive [26]. In our study, PPI use significantly increased PD risk in patients without a history of drinking or smoking. Similarly, PPI use was associated with a significantly increased PD risk in patients with a CCI score of ≥3 points compared with that in patients with a CCI score of 0, 1, or 2 points. Cigarette smoking is a well-known representative factor that plays a protective role in PD [42]. Previous studies evaluating the relationship between alcohol intake and PD risk have shown contrasting results [43, 44]. However, recent studies reported that alcohol consumption at a light to moderate level is associated with a decreased risk of PD, whereas heavy drinking is not [45, 46]. Therefore, the quantity of alcohol consumed is important for decreasing PD risk. Although measuring the exact amount of alcohol consumption by dividing alcohol consumption by the number of drinks per week is difficult, our study showed an increase in PD risk in non-drinkers. These results were consistent with those in previous studies. Biological protective components of alcohol may influence the risk of PD [47–50]. Therefore, drinking a modest amount of alcohol can be helpful and appears to have a more profound protective effect than a neurotoxic effect. SY Jung et al. also reported that PD risk was reduced in non-drinkers-turned-light drinkers, and light drinkers had an increased risk of developing PD when ceasing alcohol consumption [45]. Furthermore, they also reported that smoking and alcohol consumption may have a joint protective effect against PD; however, the optimal amount may differ by race and ethnicity. Further research is needed to elucidate the mechanism by which smoking and alcohol may interact in reducing future PD occurrence.

Our study has numerous strengths, including its large sample size and homogeneity of the study population. Furthermore, our study reflects the real-world pattern of PPI use using data from the NHIS database, which covers approximately 97% of the Korean population. Additionally, we adjusted for clinical variables that are risk factors for PD; risk behaviors, such as alcohol and smoking; and comorbidities, which were corrected using the CCI score. Moreover, we attempted to extensively exclude factors that could cause misclassification. Thus, our study provides supportive evidence for research to find possible mechanisms for the impact of PPIs on PD, which may offer insights into the pathogenesis of PD. Further research is also needed to evaluate groups at high risk for PD associated with PPI.

However, this study also has some limitations. First, although PD was defined according to the expert opinion of a neurologist and definitions from existing studies and cases with other codes that may have resulted in misclassification were excluded, we applied an operational definition of PD. Therefore, these diagnoses may be inaccurate and may lead to overdiagnosis and overestimation. Additionally, the inclusion of some Parkinsonism-like diseases may lead to bias in the results. Second, PPI use was determined by prescription claims; therefore, data regarding patient adherence to medication were unavailable. Third, we matched the patients with controls and adjusted for potential confounding factors; however, the possibility of prescription bias cannot be excluded in observational studies estimating long-term drug effects. Third, information on other confounders such as H2 blocker prescriptions and GERD that triggers PPI administration was not available for analysis in this study. Thus, future studies that examine the effect of PPI on PD risk with adjustments for those potential confounding factors would be necessary. Finally, the possibility of protopathic bias should be considered. Early PD diagnosis is necessary; however, at the time of PD diagnosis, it was difficult to determine whether PD was in the early or moderate stage. Accordingly, we applied various lag periods to reduce reverse causality bias. Furthermore, we conducted multiple regression analyses to adjust for potential confounding biases.

## Conclusion

In conclusion, our findings revealed that PPI use was associated with an increased risk of PD. Additionally, a significant positive dose-response relationship existed between the cDDDs of PPIs and PD development, and older individuals (i.e., those aged ≥50 years) were more susceptible to the PPI-related risk of PD. Thus, greater caution may be required for older patients with comorbidities to reduce this risk. Nonetheless, our findings should be interpreted with caution, and future studies to validate and further characterize our findings are warranted.

## Supporting information

S1 Table Drug codes for proton pump inhibitors and Parkinson’s disease drugs. (DOCX)

S2 Table Modified Charlson comorbidity index based on the ICD-10 code. (DOCX)

S3 Table Risk of Parkinson’s disease in individuals with PPI exposure stratified by various clinical variables. (DOCX)
