# Supplementary material for: Potential risk of proton pump inhibitors for Parkinson’s disease: A nationwide nested case-control study
Source: PLoS One. 2023 Dec 14;18(12):e0295981. doi: 10.1371/journal.pone.0295981 (PMC10721081; doi:10.1371/journal.pone.0295981)
Supplement: S1 Table — (DOCX) [file pone.0295981.s001.docx]

### S1 Table. Drug codes for proton pump inhibitors and Parkinson’s disease drugs

| **Drug** | **Main ingredient code** | **Active ingredient and dosage** |
| --- | --- | --- |
| **PPI** | 505501ATE | Ilaprazole 10 mg |
|  | 204403ATE | Omeprazole 10 mg |
|  | 519202ATE | S-Pantoprazole sodium trihydrate (as S-pantoprazole 10 mg) |
|  | 222201ATB | Rabeprazole sodium 10 mg |
|  | 222201ATE | Rabeprazole sodium 10 mg |
|  | 181302ATD | Lansoprazole 15 mg |
|  | 181302ATE | Lansoprazole 15 mg |
|  | 181302ACE | Lansoprazole granule (as lansoprazole 15 mg) |
|  | 181302ACH | Lansoprazole 15 mg |
|  | 367201ATD | Esomeprazole magnesium (as esomeprazole 20 mg) |
|  | 498001ACH | Esomeprazole strontium tetrahydrate (as esomeprazole 20 mg) |
|  | 509902ACH | Esomeprazole 20 mg |
|  | 519201ATE | S-Pantoprazole sodium trihydrate (as S-pantoprazole 20 mg) |
|  | 367201ACH | Esomeprazole magnesium (as esomeprazole 20 mg) |
|  | 204401ATE | Omeprazole 20 mg |
|  | 208802ATE | Pantoprazole sodium (as pantoprazole 20 mg) |
|  | 208901ATE | Pantoprazole sodium sesquihydrate (as pantoprazole) 20 mg |
|  | 204401ACE | Omeprazole granule (enteric coated) (as omeprazole 20 mg) |
|  | 367201ATB | Esomeprazole magnesium (as esomeprazole 20 mg) |
|  | 222202ATE | Rabeprazole sodium 20 mg |
|  | 621901ACR | Dexlansoprazole 30 mg |
|  | 181301ATD | Lansoprazole 30 mg |
|  | 181301ATB | Lansoprazole 30 mg |
|  | 181301ACE | Lansoprazole granule (as lansoprazole 30 mg) |
|  | 181301ATE | Lansoprazole 30 mg |
|  | 181301ACH | Lansoprazole 30 mg |
|  | 656701ATE | Pantoprazole hemimagnesium monohydrate (as pantoprazole 40 mg) |
|  | 519203ATE | S-Pantoprazole sodium trihydrate (as S-pantoprazole 40 mg) |
|  | 367202ATD | Esomeprazole magnesium (as esomeprazole 40 mg) |
|  | 498002ACH | Esomeprazole strontium tetrahydrate (as esomeprazole 40 mg) |
|  | 204402ATE | Omeprazole 40 mg |
|  | 509901ACH | Esomeprazole 40 mg |
|  | 367202ACH | Esomeprazole magnesium (as esomeprazole 40 mg) |
|  | 208801ATE | Pantoprazole sodium (as pantoprazole 40 mg) |
|  | 208902ATE | Pantoprazole sodium sesquihydrate (as pantoprazole 40 mg) |
|  | 367202ATB | Esomeprazole magnesium (as esomeprazole 40 mg) |
|  | 621902ACR | Dexlansoprazole 60 mg |
| **Anti-Parkinson’s disease** | 625201ATB | Rasagiline mesylate 1.56 mg (as rasagiline 1 mg) |
|  | 507300ATB | Levodopa 125 mg |
|  | 507200ATB | Levodopa 75 mg |
|  | 499900ATB | Levodopa 200 mg |
|  | 468900ATB | Levodopa 150 mg |
|  | 468400ATB | Levodopa 50 mg |
|  | 468300ATB | Levodopa 100 mg |
|  | 439201ATB | Entacapone 200 mg |
|  | 402507ATR | Pramipexole dihydrochloride 1.5 mg |
|  | 402506ATR | Pramipexole dihydrochloride 0.75 mg |
|  | 402505ATR | Pramipexole dihydrochloride 0.375 mg |
|  | 402504ATB | Pramipexole dihydrochloride 1 mg |
|  | 402503ATB | Pramipexole dihydrochloride 0.5 mg |
|  | 402502ATB | Pramipexole dihydrochloride 0.25 mg |
|  | 402501ATB | Pramipexole dihydrochloride 0.125 mg |
|  | 256500ATR | Levodopa 200 mg |
|  | 256400ATB | Levodopa 250 mg |
|  | 256300ATB | Levodopa 100 mg |
|  | 256200ATB | Levodopa 200 mg |
|  | 256000ATR | Levodopa 100 mg |
|  | 256000ATD | Levodopa 100 mg |
|  | 256000ATB | Levodopa 100 mg |
|  | 226401ATB | Selegiline hydrochloride 5 mg |
|  | 224906ATR | Ropinirole hydrochloride (as ropinirole 8 mg) |
|  | 224905ATR | Ropinirole hydrochloride (as ropinirole 4 mg) |
|  | 224904ATB | Ropinirole hydrochloride (as ropinirole 5 mg) |
|  | 224903ATB | Ropinirole hydrochloride (as ropinirole 0.25 mg) |
|  | 224902ATR | Ropinirole hydrochloride (as ropinirole 2 mg) |
|  | 224902ATB | Ropinirole hydrochloride (as ropinirole 2 mg) |
|  | 224901ATB | Ropinirole hydrochloride (as ropinirole 1 mg) |
|  |  |  |

Abbreviations: PPI, proton pump inhibitor

* drugs that can be used for indications other than PD, including amantadine, apomorphine, benzatropine, biperiden, bromocriptine, cabergoline, dihydroergocryptine, orphenadrine, piribedil, procyclidine, rotigotine, and trihexyphenidyl, were excluded.
