# Supplementary material for: Potential risk of proton pump inhibitors for Parkinson’s disease: A nationwide nested case-control study
Source: PLoS One. 2023 Dec 14;18(12):e0295981. doi: 10.1371/journal.pone.0295981 (PMC10721081; doi:10.1371/journal.pone.0295981)
Supplement: S2 Table — (DOCX) [file pone.0295981.s002.docx]

### S2 Table. Modified Charlson Comorbidity Index based on the ICD-10 code

| **Disease** | **Weight** | **ICD-10 code** |
| --- | --- | --- |
| Myocardial infarction | 1 | I21x, I22x, I252 |
| Congestive heart failure | 1 | I099, I110, I130, I132, I255, I420, I425–I429, I43x, I50x, I290 |
| Peripheral vascular disease | 1 | I70x, I71x, I731, I738, I739, I771, I790, I792, K551, K558, K559, Z958, Z959 |
| Cerebral vascular accident | 1 | G45x, G48x, H340, I60x–I69x |
| Dementia | 1 | F00x–F03x, F051, G30x, G311 |
| Chronic pulmonary disease | 1 | I278, I279, J40x–J47x, J60x–J67x, J684, J701, J703 |
| Rheumatologic disease | 1 | M05x, M06x, M315, M32x–M34x, M351, M353, M360 |
| Peptic ulcer disease | 1 | K25x–K28x |
| Mild liver disease | 1 | B18x, K700–K703, K709, K713–K715, K717, K73x, K74x, K760, K762–K764, K768, K769, Z944 |
| Diabetes without chronic complication | 1 | E100, E101, E106, E108, E109, E110, E111, E116, E118, E119, E120, E121, E126, E128, E129, E130, E131, E136, E138, E139, E140, E141, E146, E148, E149 |
| Diabetes with chronic complication | 2 | E102–E105, E107, E112–E115, E117, E122–E125, E127, E132–E135, E137, E142–E145, E147 |
| Hemiplegia or paraplegia | 2 | G041, G114, G801, G802, G81x, G82x, G830–G834, G839 |
| Renal disease | 2 | I120, I131, N032–N037, N052–N057, N18x, N19x, N250, Z490–Z492, Z992 |
| Any malignancy, including leukemia and lymphoma | 2 | C00x–C26x, C30x–C34x, C37x–C41x, C43x, C45x–C58x, C60x–C76x, C81x–C85x, C88x, C90x–C97x |
| Moderate or severe liver disease | 3 | I850, I859, I864, I982, K704, K711, K721, K729, K765, K766, K767 |
| Metastatic solid tumor | 6 | C77x – C80x |
| Acquired immune deficiency syndrome/HIV | 6 | B20x–B22x, B24x |
|  |  |  |
