# Supplementary material for: Potential risk of proton pump inhibitors for Parkinson’s disease: A nationwide nested case-control study
Source: PLoS One. 2023 Dec 14;18(12):e0295981. doi: 10.1371/journal.pone.0295981 (PMC10721081; doi:10.1371/journal.pone.0295981)
Supplement: S3 Table — (DOCX) [file pone.0295981.s003.docx]

### S3 Table. Risk of Parkinson’s disease in individuals with PPI exposure stratified by various clinical variables

| **Variable** | **N** | **Including a lag window of 1 year** | | **Including a lag window of 2 years** | | **Including a lag window of 3 years** |
| --- | --- | --- | --- | --- | --- | --- |
|  |  | **Adjusted OR (95% CI)†** |  | **Adjusted OR (95% CI)†** |  | **Adjusted OR (95% CI)†** |
| Age group |  |  |  |  |  |  |
| <50 years | 5,550 | 1.11 (0.93–1.31) |  | 1.04 (0.87–1.25) |  | 0.98 (0.79–1.20) |
| ≥50 years | 23,955 | 1.10 (1.07–1.13)*** |  | 1.11 (1.07–1.14)*** |  | 1.10 (1.06–1.13)*** |
| Sex |  |  |  |  |  |  |
| Male | 69,799 | 1.06 (1.01–1.11)* |  | 1.07 (1.02–1.12)** |  | 1.04 (0.99–1.10) |
| Female | 86,830 | 1.13 (1.09–1.18)*** |  | 1.14 (1.09–1.18)*** |  | 1.13 (1.09–1.18)*** |
| BMI group (kg/m^2^) |  |  |  |  |  |  |
| Underweight (<18.5) | 4,150 | 1.31 (1.05–1.63)* |  | 1.29 (1.03–1.62)* |  | 1.30 (1.02–1.67)* |
| Normal (18.5–<25.0) | 9,569 | 1.06 (1.01–1.11)* |  | 1.08 (1.03–1.13)** |  | 1.07 (1.02–1.12)** |
| Overweight (25.0–<30.0) | 50,090 | 1.16 (1.09–1.23)*** |  | 1.15 (1.09–1.23)*** |  | 1.13 (1.06–1.21)*** |
| Obesity (≥30.0) | 6,720 | 1.49 (1.25–1.79)*** |  | 1.36 (1.14–1.63)** |  | 1.46 (1.20–1.77)*** |
| HTN |  |  |  |  |  |  |
| No | 155,630 | 1.09 (1.06–1.13)*** |  | 1.10 (1.07–1.14)*** |  | 1.09 (1.05–1.13)*** |
| Yes | 1,000 | 16.97 (7.76–37.11)*** |  | 5.02 (2.62–9.62)*** |  | 7.68 (3.54–16.65)*** |
| DM |  |  |  |  |  |  |
| No | 156,290 | 1.10 (1.07–1.13)*** |  | 1.10 (1.07–1.14)*** |  | 1.09 (1.06–1.13)*** |
| Yes | 340 | 3.78531e+19 (0.00–Inf) |  | 3.08 (0.25–38.34) |  | 5.11 (0.32–82.72) |
| CCI score group |  |  |  |  |  |  |
| 0 | 65,350 | 1.05 (0.98–1.13) |  | 1.05 (0.97–1.13) |  | 1.03 (0.95–1.13) |
| 1 | 46,386 | 1.03 (0.95–1.12) |  | 1.03 (0.94–1.12) |  | 1.01 (0.92–1.10) |
| 2 | 24,364 | 1.06 (0.92–1.21) |  | 1.05 (0.91–1.20) |  | 1.14 (0.99–1.33) |
| ≥3 | 20,529 | 1.29 (1.10–1.52)** |  | 1.31 (1.12–1.53)** |  | 1.38 (1.17–1.61)*** |
| Smoking status |  |  |  |  |  |  |
| Never | 111,611 | 1.11 (1.07–1.15)*** |  | 1.11 (1.07–1.16)*** |  | 1.12 (1.07–1.16)*** |
| Ex/Current | 62,869 | 1.22 (1.04–1.44)*** |  | 1.15 (0.97–1.36) |  | 1.08 (0.91–1.30) |
| Alcohol consumption (times/week) | |  |  |  |  |  |
| None | 114,274 | 1.12 (1.08–1.17)*** |  | 1.14 (1.10–1.18)*** |  | 1.13 (1.08–1.17)*** |
| 1–2 | 25,554 | 1.01 (0.87–1.17) |  | 0.97 (0.84–1.13) |  | 0.93 (0.79–1.09) |
| ≥3 | 16,801 | 1.17 (0.93–1.46) |  | 1.04 (0.83–1.30) |  | 0.94 (0.73–1.20) |

Abbreviations: BMI, body mass index; CCI, Charlson Comorbidity Index; CI, confidence interval; DM, diabetes mellitus; HTN, hypertension; OR, odds ratio; PPI, proton pump inhibitor
The odds ratio (OR) and 95% confidence intervals (CIs) were computed from the conditional logistic regression model.
† Adjusted for the calendar year of the index date, CCI score, smoking status, and alcohol consumption.
*p<0.05; **p<0.01; ***p<0.001
